# Firm value in the airline industry: perspectives on the impact of sustainability and Covid-19

**DOI:** 10.1057/s41599-023-01644-8

**Published:** 2023-06-06

**Authors:** Yaghoub Abdi, Xiaoni Li, Xavier Càmara-Turull

**Affiliations:** Department of Business Management, Universitat Rovira i Virgili Reus, Tarragona, Spain

**Keywords:** Business and management, Finance

## Abstract

To date, there has been limited research undertaken into firm value determinants in the air transport industry, one of the most essential sectors for global business. In view of this, in this study, we review and synthesise the literature that focuses on the value of firms in this sector and discuss conceptually and empirically the determinants influencing airlines’ stock values. Our main objective is to widen our understanding of the current state of research on the firm value of air transport companies. Using the systematic literature review (SLR) approach, we classify 173 papers published from 1984 to 2021. We find considerable changes in academic interest in the topic over the time period analysed, especially as a consequence of crisis-induced market crashes. In addition, we classify the main research themes relating to airlines’ market value, identify gaps, and introduce potential future research avenues in this area. Among the themes identified, the adjustment in the industry-level factors such as alliances, market structure and competition were the most common source of fluctuations in airlines’ stock value. However, we find shifting to sustainability initiatives and its consequence for stakeholders’ value as one of the most discussed topics in this context. The trend has gained attention since early 2020 due to the emergence of the Covid-19 pandemic as companies are looking for green and sustainable ways to protect the value in crisis time. Our findings assist transportation researchers and executives in addressing major value drivers of airline firms.

## Introduction

The airline industry is arguably among the world’s most widespread, fastest growing and rapidly rising industries, providing a wide variety of services for people worldwide (Belobaba et al., 2009). In recent years, air transport has become one of the primary modes of travel. This is confirmed by the International Civil Aviation Organisation (ICAO), which reported that in 2019 the number of air travellers rose to 4.5 billion (ICAO, 2019). While the industry was hit hard by the COVID-19 pandemic, suffering dramatic falls in the rate of the services provided and passenger numbers, there are evidence that the industry is recovering (McKinsey, 2021). According to the International Air Transport Association (IATA), in 2021 the overall figure was 47% of the number of passengers in 2019 (IATA, 2022), with expected increases to 83% in 2022, 94% in 2023, 103% in 2024 and 111% in 2025. Therefore, a continuation of this growth rate would mean passenger demand doubling over the next years.

Since its establishment, many aspects of air transport have undergone structural reforms involving technological advances (e.g., the emergence of commercial jet aircraft in the 1950s and the design of wide-body jumbo jets in the 1970s), administrative procedures (e.g., the deregulation process starting in the US in 1978) and financial aspects (the continual growth of airline firms listed in various stock exchanges on a global scale) (Belobaba et al., 2009; Malighetti et al., 2011). The trend follows the general acceleration of supply chain companies to gain a competitive advantage (Azadian, 2020). These changes have re-shaped the industry, altering previously fundamental characteristics and markedly changing perspectives in the industry, leading to an environment of market competition (Cook, 1996). Since starting to finance their operations from stock markets rather than relying on state support, issues such as the use of financial analyses on stock returns, cost-of-capital, and the valuation of assets and securities have become crucial (Malighetti et al., 2011). In this regard, the industry has been transformed into a much riskier one (Vasigh et al., 2020). To remain efficient, it is clear that air carriers must seriously consider financial management and the economic environment in which they operate.

Given that the ultimate objective of financial management for today’s value-minded executives is value creation and maximising shareholders’ profits (McKinsey, 2020), firm value has become an important consideration in stakeholders’ financial attitudes. Market value offers meaningful insight into the valuation of a company and can assist both airline executives and investors to determine the airline’s financial status. Due to the importance of this parameter, studies have focused on several factors affecting the value of airlines. However, a review of the literature shows that academics have paid insufficient attention to valuation studies (Malighetti et al., 2011). While many reviews have been carried out in the air transport context (Matias Ginieis et al., 2011, 2012; Khudhair et al., 2019; Mardani et al., 2015; Kaps and Phillips, 2017; Kalemba and Campa-Planas, 2011; Campa-Planas and Kalemba, 2017; Spasojevic et al., 2018; Wang and Gao, 2021; Sun et al., 2021; Duval, 2013; Raza et al., 2020; Papatheodorou, 2021), no comprehensive and generic review of the contextual factors that affect a firm’s valuation has yet been carried out.

An analysis of academic contributions, however, shows that firm value has often been linked to different business strategies and policies, and is thereby influenced by several factors. This poses the question as to what the value determinants are and how they can be classified. The systematic review conducted by Pereira et al. (2021) appears to be the only study to map out the value-creating business activities in the aviation industry. The authors identified 114 value-creating innovations and how they add value to the industry. Based on the results, initiatives relating to efficiency, convenience, portfolio differentiation and sustainability stood out. Taking a more comprehensive overview, the present study spans determinants such as firm and industry level factors, health crises, political and economic stability, customer relationship, etc. as value drivers to identify the content relationships and approaches, and to explore the gaps in the literature. This topic is extremely important given that the COVID-19 pandemic plunged the entire industry into crisis and destroyed stakeholder value, making it crucial to fill any gaps. We theoretically document the concept of firm value, then gather related studies, commenting on the factors involved, which are grouped into corresponding themes. This approach enabled us to unpack the nature of drivers of firm value and to recognise how these elements have been represented in academic contributions, finally indicating future research directions. From this perspective, the present study sought to answer the following questions:

- How has the literature on firm value evolved in the air transport industry?
- What are the main research directions relating to firm value in the industry?
- What are the main influential factors of firm value in the industry?
- In what context has the research focus shifted from traditional value drivers to sustainability initiatives?
- What are the niches for forthcoming academic investigations on this topic?

The current study answers the aforementioned study questions by means of the systematic literature review or SLR method, and via thematic scrutiny of the relevant information, using the WoS Core Collection-Clarivate & Scopus databases. There is limited documentation on review studies in the field of air transportation, with existing reviews able to be divided into two streams. First are the articles focusing on the theoretical and empirical study of the air transportation domain. They notably consider specific factors such as air travel demand (Wang and Gao, 2021), revenue management (Raza et al., 2020) and service quality (Kalemba and Campa-Planas, 2011) and safety (Campa-Planas and Kalemba, 2017), or they organise the study around the COVID-19 pandemic (Sun et al. 2021). Other studies revolve around reviewing the interrelationship between the airline industry and tourism (Papatheodorou, 2021), and the role of airlines in economic development (Lenaerts et al., 2021).

The aim of our review was to systematically identify and discuss the progress of academic research into firm value in the air transport context, including how this has changed over time in terms of the number of papers focusing on the topic, the host (journals) for publication, and the most productive researchers, countries, etc. The aim of this investigation was to provide a multidisciplinary analysis of the ongoing discussion on firm value in the airline sector by building bridges among different perspectives and identifying the factors impacting firm value. In addition, by mapping out these value drivers we could establish a broad discussion of problem-based solutions to the construction of knowledge from the management perspective. A summary of the identified gaps in the literature, the research questions, and the contribution of the present study is presented in Table 1.

**Table 1:** Summarising the study’s contribution and aims.

Research gaps	Research questions	Contributions
Although market value is an important factor delivering insight of firm’s financial position and health, there is no overview of how it has been reflected amongst academics.	What firm value determinants have been explored in the previous scholarly works for companies operating in the air transport industry?	To show the evolution of published papers, determine the co-authorship among the main researchers in this field, identify authors, countries and journals working in this area.
There is a lack of structured thematic understanding of factors influencing firm value in air transport industry	What are the main research directions of the literature related to firm value in this industry?	To obtain the knowledge structure and hotspots of the research field (by designing keyword co-occurrence network) with the help of bibliometric analysis, identify main research themes in which firm value has been reflected.
Further, there is no study considers the evolution of value drivers during the time.	What value drivers have been on the spotlight during the time?	To show the thematic evolution of published papers during the time. We investigate how academic interest has changed in focus on value drivers. Recently, sustainability issues have been on spotlight. We follow the direction at the industry.
There is a lack of structured thematic understanding of factors influencing firm value in air transport industry.	What are the niches for future researchers to explore in this area?	To present and verify the necessity to address a research problem and to suggest the pragmatic methods in which the future investigation should be carried out, to develop new tests and processes that could eventually help for obtain more comprehensive framework of knowledge of the area for the theory and practitioners.

The paper is structured as follows. Section “State of the art” presents a theoretical framework of the main research; the section “Method” describes the research methodology, sample selection and search strategy; and the section “Assessment of the selected publications” reports the results of the review. The paper ends with some conclusions, policy reflections and research limitations.

## State of the art

This section briefly reviews the business environment of airlines, the concept of a firm and its internal value factors, as well as external factors influencing market value.

### Airline sector

The global aviation industry is a worldwide service provider and has a fundamental responsibility in the establishment of the world’s economy (Belobaba et al., 2009). The industry assumes this role through its activities and impact on related industries. Airlines receive a huge volume of funds and recruit thousands of people, all of which contribute to innovation and economic growth. Air transport is also a driver of globalisation. It improves standards of living by widening leisure and cultural experiences for the people, helps to enhance living standards, and advances sustainability via facilitating tourism and trade. The development of air transport and its technical advancements and service performance make it one of the major means of building of modern world (ICAO, 2017).

**Fig. 1:**
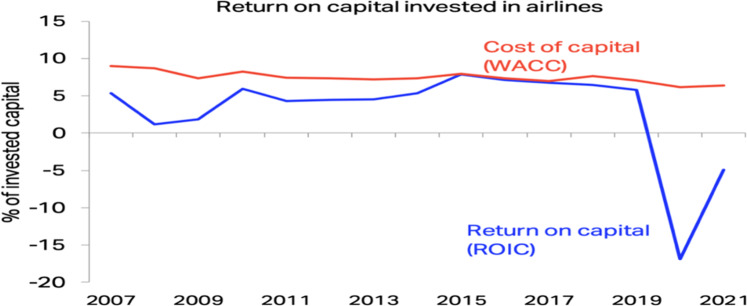
Median ROIC vs. WACC of the airline industry (IATA Economics, 2020). Return on capital invested in the wole airlines industry was lower than cost of capital.

In order to improve the performance of the industry, it is critically important for airline companies to operate efficiently, the most efficient ones can usually offer lower prices and consequently attract more passengers (Assaf and Josiassen, 2012). In addition, more efficient companies can use the benefits of scale by having a larger number of travellers which contributes to better global recognition and image for their brand. Traditionally, air transport has generated some of the lowest returns across business sectors. According to the 2020 year-end economic report by the international air transport association (IATA Economics, 2020), even before the current pandemic crisis, equity holders had failed to gain an adequate return for their finances. As shown in Fig. 1, IATA documents the divergence of returns on invested capital (ROIC) against the weighted average cost of capital (WACC), for the years 2007–2021. The function of ROIC is to measure the financial efficiency of a firm in which how well the invested capital under its control has been allocated to produce profitable investments. It also shows how well a company’s money has been utilised to generate revenue (Lee, 2019). One can see that the companies operating in this sector have rarely generated revenues as high as the WACC for the industry as a whole.

### Firm value

Market value is an attempt to estimate the value of a property under open market conditions (Pagourtzi et al., 2003). In other words, it refers to the price of an asset at which a supplier and a buyer would agree to change its ownership. An agreed market value satisfies both the seller and the buyer and usually refers to the stock price of publicly listed trading companies. Stock price variation, therefore, represents a percentage change in a firm’s market value at any given time and is driven by supply and demand. Market value is estimated by applying valuation methods that reflect the nature of the asset and the underlying environment in which it could be traded in trade markets (Pagourtzi et al., 2003).

Book value is an accounting concept that measures the value of a firm using assets as they are recorded on a balance sheet. It represents the wealth of a company in assets as well as the value of the company’s stockholder equity, as registered on a balance sheet. Book value is considered the sum of a firm’s historical records of assets and liabilities together with documented costs and revenues carried forward to future periods (Boulton et al., 2003). Investors are particularly interested in the association between market and book value, considering shares selling well above the book value a target for overvalued portfolios, and those selling below it as undervalued portfolios. Market value and book value together help the company to produce insights into its business prospects. However, due to its ability to instantly reflect the growth or collapse of a firm, the market value provides a better indication of investors’ expectations regarding its business prospects. Some specific applications and issues are addressed using the book-to-market ratio, which compares the original cost of the asset and the firm’s market value as calculated by its market capitalisation. It is an important firm-level indicator of a company’s returns, irrespective of size and the geographical location in which it is operating (Cakici and Topyan, 2014). It dramatically highlights any growing discrepancies between book value and market capitalisation (Boulton et al., 2003).

Asset valuation theories have long been of interest to both investors and academics in finance. The literature on firm valuation highlights the discrepancy between a firm’s market value and its book value by means of the present value of future abnormal earnings. In this context, book-to-market value (B/M) reflects the investors’ estimate of a firm’s abnormal earnings. The Fama-French (1996) three-factor model argues that a large proportion of the discussed CAPM average-return anomalies in the literature such as size, book-to-market ratio, earnings/price, cash flow/price and past sales growth are related, and their model is capable of addressing them. Size, book-to-market ratio and excess return on the market are the three main elements of the model introduced by Fama-French. These factors are used as *small minus big* (SMB), *high minus low* (HML) and the return of the portfolio minus the risk-free rate of return. The same authors also introduced the size effect and the B/M effect as two behavioural anomalies. These two effects argue that small firm stock tends to have a higher return than large firm stock, and that firms with a high B/M (when the market value is significantly lower than the book value) tend to have continuously low earnings, respectively. Meanwhile, a low B/M (when the market value is significantly higher than the book value) is a signal for sustainable profit growth (Fama and French, 1995). In other words, a negative difference between stock price and book value signals a potential impairment, specifically when the discrepancy exists for a long period (Bini and Penman, 2013). Conversely, when the stock price exceeds the book value, it can be assumed that the firm is able to generate revenues, or its stocks have a higher market value.

This issue has been discussed in the associated literature, which reveals that calls were made to reform accounting standards, the conventional historical cost approach having outlived its usefulness (Boulton et al., 2003). These calls resulted in a transition from an industrial to a fundamentally knowledge-based approach. Based on this method, intangible assets are considered the new drivers of economic activity (Skinner, 2008; Canibano et al., 2000). The valuation of intangible assets has become a significant contemporary discussion point for researchers in different fields of human knowledge in their attempt to identify relevant intangibles for management purposes and firm valuation (Fazzini, 2018; Lim et al., 2020; García-Ayuso, 2003). In this regard, the OECD (2012) argues that in the studies on this topic, better management is positively correlated with the disclosure of intangibles and financial performance.

### Internal factors influencing firm value of airlines

**Fig. 2:**
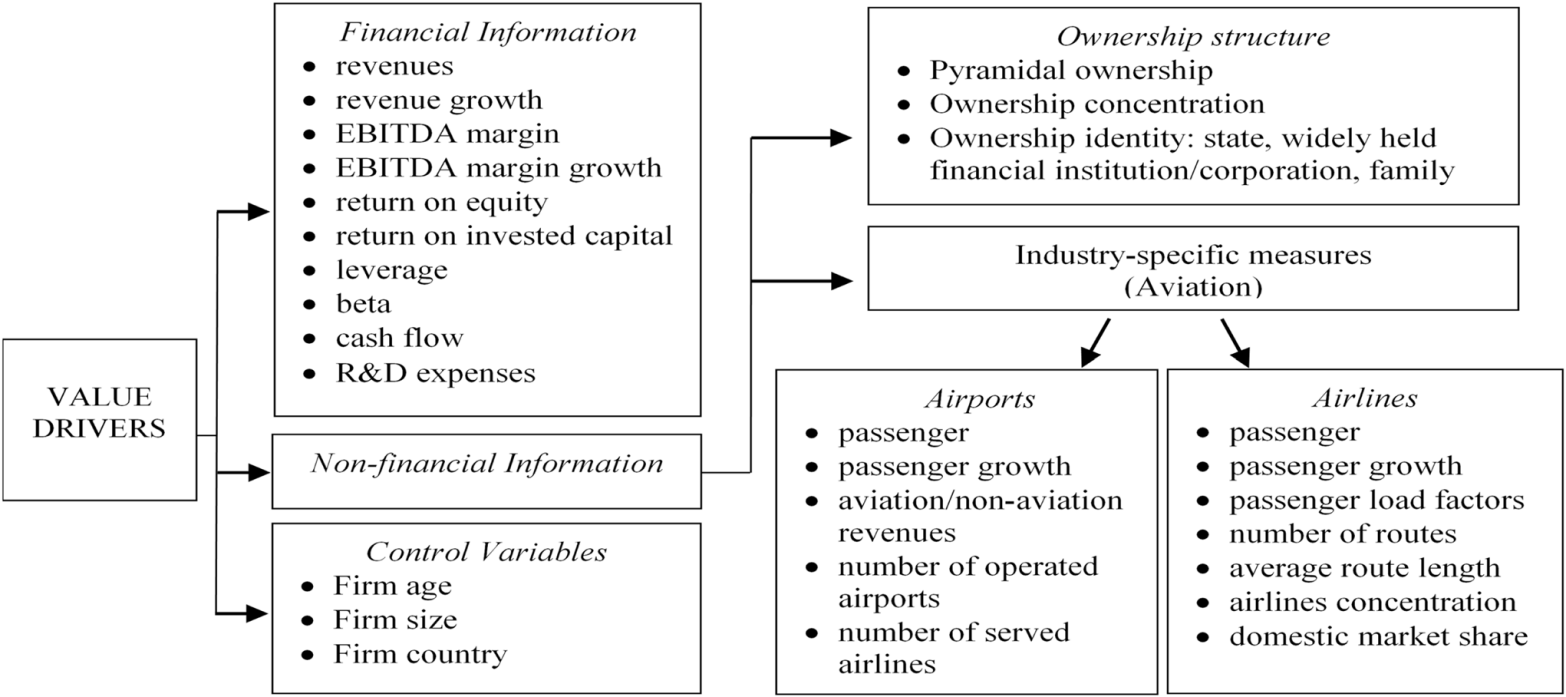
Value drivers at the air transport industry based on Malighetti et al. (2011). Internal annd external factors influencing market value of airlines.

Works by Li et al. (2004) and Malighetti et al. (2011) investigate the industry-level value determinants for airlines. Both studies suggest a range of possible determinants influencing firm value. For their part, Malighetti et al. (2011) collected data from 87 airlines and 24 airport companies to test the value relevancy of a broad range of variables, as summarised in Fig. 2, showing that many internal factors including the above-explained items affect firm value. The variables considered were categorised into four potential value determinants to regress against firm value. The four value driver categories provided information about the financial status of the firm, the type of ownership, and industry-specific and control variables. Drivers of shareholder wealth were introduced into this framework, based on extensive previous literature highlighting the structure of the market, the role played by the network (e.g., flight frequency, size of the aircraft, number of routes under competition, and market share on both local and worldwide scales) and the type of business model (i.e., low-cost, or full-service) chosen by the airline. They found that the ownership structure has a direct relationship with firm value: a higher degree of ownership concentration is associated with a higher market value. This is probably due to the greater tendency to maximise firm value under these conditions. This outcome is consistent with the general industry view asserting a positive link between state ownership and both efficiency and return for firms operating with higher levels of debt and a higher equity ratio (Le and O’Brien, 2010). It also concurs with the results of the research conducted in this paper since the airline industry is a capital-intensive sector with a high debt-to-equity ratio. Conversely, the study found that firm size and age are negatively correlated with the firm value of airlines.

### External factors influencing firm value of airlines

The aviation industry is extremely sensitive to external economic, political, and social factors due to its heavy dependency on a wide range of business and industrial support. Government policies, regulations, mass media, and customers are parties having an impact on airlines’ market value. Due to their significance in terms of these factors and particularly due to bilateral trade agreements (Haanappel, 1980), the majority of international airlines (except in the United States) were in control of governments until the mid-1980s when this type of airline was deemed to be the best model ensuring the growth of industry (Belobaba et al., 2009). Therefore, the industry is highly connected with politics and government elites. Almost all state-owned air carriers suffer from *distressed state airline syndrome* which is a political and organisational virus affecting this type of airline due to issues such as substantial losses (e.g. large volume of collected debts, undercapitalised and indirect subsidies that hide real losses), over-politicised, bureaucratic management, poor service quality, etc. (Doganis, 2001).

A sizeable portion of the industry involves cross-border operations so that not only the domestic market but also economic conditions around the globe, affect the industry’s performance. In this regard, academic research has widely reflected the issues and claims that establishing strategies to utilise their firm-level strengths and neutralise external weaknesses may build sustained competitive advantage (Porter, 1985; Song et al., 2021). In this regard, the term *contagion* or *herd behaviour* is used to describe the transmission of instability or unexpected phenomena in one industry(country) to another because of trade, financial or other economic linkages between them (Gillen and Lall, 2003). A wide range of disasters, terror attacks, earthquakes, and aircraft crashes are highlighted in the literature as having implications for firms’ financial decision-making (Fernandez-Perez et al., 2021).

Studies also considered the consequences of health crises like the SARS and the current Covid-19 pandemic on firm performance. Such outbreaks challenge health care, economic, and financial systems worldwide. The problem is more severe for the airline industry because the shutdown significantly restricts people’s movement decreasing passenger demand for flights. Financial market uncertainty will be triggered by negative sentiment in the operation and business environments. Therefore, airlines lose their value in global financial markets. To summarise, this study categorises a variety of factors that changed considerably and have implications on the value of the firm. These factors range from political, environmental and social factors to the public health situation.

## Method

### SLR

Systematic review is a methodology that finds existing studies, picks contributions, analyses and synthesises data, and reports to reach out clear conclusions about existing knowledge and unidentified aspects of those publications (Denyer and Tranfield, 2009). Alternatively, according to the document published by the Centre for Reviews and Dissemination (CRD), the method aims to identify, assess and outlines the findings of all relevant research papers to facilitate the accessibility of available evidence to policy-makers (CRD, 2009). It also serves in two fundamental ways; identifying gaps to suggest future research avenues and providing key information as a framework (Kitchenham, 2004). Therefore, reviewing the literature is an important part of every research topic. Moreover, a profound understanding of the necessary processes and skills and owing experience in the respective field are compulsory to conduct a literature review (Fisch and Block, 2018).

Based on the work by Tranfield et al. (2003), the systematic literature review method helps to avoid the biases of traditional reviews of the current body of literature. This method allows the researcher to summarise the to-date literature relevant to the topic of interest; analyses the topic from various perspectives; and, finally, provide reliable insight from a pool of knowledge dispersed across a broad range of studies. SLR could be very useful to evaluate available information and subsequent understanding in responding to the research objective (Kitchenham, 2004). In this study, based on the guidelines defined in CRD (2009) and Denyer and Tranfield (2009), the method was applied to provide a reference on international academic research related to firm valuation in the air transport industry.

### Search strategy

We followed three steps to execute the SLR. The first was to set up keywords and perspective combinations of those keywords in the search. Inclusion and exclusion criteria for papers found constitute a second phase to adjust the relevance of each study to the current research paper concern. In this phase, we further evaluated selected papers based on certain characteristics. Finally, meta-analysis of selected papers, such as year-wise distribution of selected studies, identifying the most productive author, geographical setting, and co-authorship maps as well as keyword co-occurrence analysis, was a third step. We further provide a thematic analysis of sampled articles to elucidate the main research strands in the field.

#### Databases and software

**Fig. 3:**
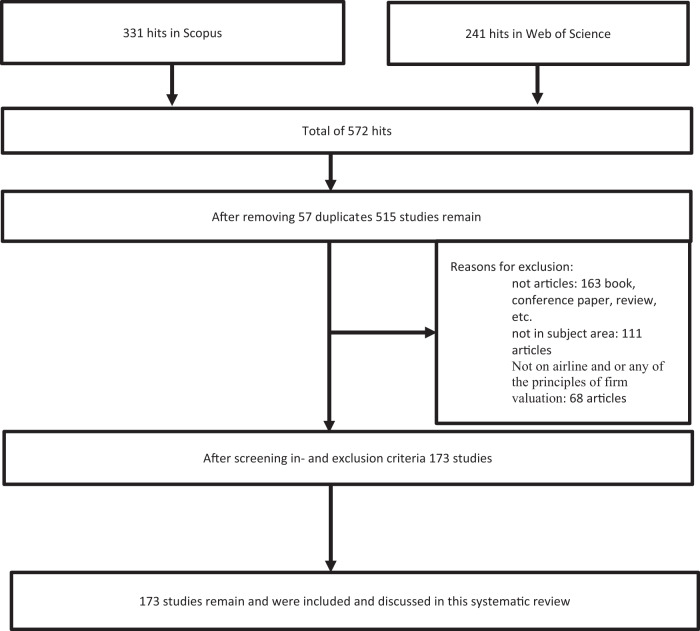
Research process summary. Our initial result of 572 documents was filtered by including only peer-reviewed articles and those related to the research theme at the airline context, resulting in 173 eligible documents. (author created).

To carry out the review, we used documents published in journals indexed in Scopus and WoS Core Collection-Clarivate. Both databases are very well known in academia and host thousands of contributions annually. Specifically, we selected Scopus to apply keyword combinations since it is considered a large abstract and citation database for peer-reviewed literature. According to Elsevier’s website (2021), Scopus provides extensive citation search results and updates scholars and institution profiles automatically, creating a rich connection between researchers, released ideas and institutions. Clarivate Analytics’ Web of Science is also one of the most important databases, offering comprehensive citation search and analytical information tools. It holds a prominent position in its association with scientific products and features across different knowledge domains (Li et al., 2017). Consequently, both databases are useful tools to conduct systematic reviews (Campa-Planas and Kalemba, 2017; Calatayud et al., 2016; Li et al., 2017; Bergiante et al., 2015). Using the R 3.6.0 programming package, Vosviewer and Microsoft Excel, we analysed the sampled publications to determine the evolution in the published papers over the period. Figure 3 summarises the search process.

#### Keyword identification and sampling

As a search strategy highly contributes to a methodical extraction of papers, it is critical to determine which terms to use in the process of searching to find relevant articles and to determine how these will be specified during the strategy. The approach undertaken is based on the main research questions to encompass potentially relevant academic contributions. Particularly, based on the background reading and subject heading brainstorming, we selected the relevant keywords to ensure that the search is comprehensive considering different spelling, synonyms, variants of keywords and related concepts. As shown in Table 2, we used a total of eight keywords to develop the search strings: (*book value*, *market value*, *firm value*, *stock market*, *valuation*, *air transport*, *airline*, and *aviation*). These keywords were formulated to run in both databases as: “book value” OR “market value” OR “firm value” OR “stock market” OR “valuation*”) AND (“air transport*” OR “airline” OR “aviation”. In this formula, based on the guideline by Gu and Lago (2009) the Boolean operators of OR/AND have been used between keywords to allow synonyms and to link two clusters of terms, respectively. Also, to extend the range of possible studies the study uses an asterisk at the end of some keywords, somewhat different keywords for the same concept are used in some studies (Wilding et al., 2012). The aim of the phase was to retrieve articles having the most relevant keywords in their title, abstract, or keyword sections for further assessment of eligibility and inclusion.

**Table 2:** Word combination for the searching.

Book value *OR* Market value *OR* *Firm Value* *OR* Stock Market OR Valuation*	*AND*	Air transport *OR* Airline OR Aviation

#### Inclusion and exclusion criteria

In this study, we used the following criteria to select relevant articles among those found in the review and filtered any non-compliant studies out of the sample. The first criterion was that the article be published following a peer-reviewed process. Therefore, we eliminated publication forms such as book series, conference proceedings, book reviews and working papers. The second criterion was that the sampled studies had to investigate the book value or market value of one or more airlines. Next, we filtered for only articles with editorial lines related to research areas: business economics, economics, business, transportation, business finance, management, and hospitality leisure sport tourism in WoS together with including articles within business and economics subject areas in Scopus. We also included articles in the fields of environmental studies and hospitality leisure tourism to cover work related to sustainability and its effect on firm performance and the value of airlines in the WoS Core Collection-Clarivate database. It is worth noting that we did not control other potential factors such as open or closed access, year, language, etc when searching in neither WoS nor Scopus databases. This means that all studies, having any firm valuation or performance measures appearing as a search result of the above-defined keywords and consistent with the criteria, are included in the analysis. The final step was to merge both file results in the biblioshiny package (using R-Studio), after removing duplicates to achieve the final sample. The review identified a total of 572 empirical studies as follows: 411 peer-reviewed, 98 conference papers, 10 conference reviews, 16 reviews,16 books and book chapters, and a further 21 documents classified as early access (9), short survey (6), note (2), business article (1), preprints (1), and erratum (1). Contributions that failed to satisfy the inclusion criteria, such as the conference papers and the book chapters, were filtered at this stage. The strategy yielded a final sample of 173 articles.

## Assessment of the selected publications

The assessment of the selected articles is divided into two sections: a descriptive analysis and a thematic analysis. The former provides a quantitative description, summarising the features gleaned from the information obtained regarding the performance of authors and countries, year of publication and a keyword co-occurrence network. For its part, the thematic analysis emphasised the identification and interpretation of the organisation of the studies in the sampled articles based on the similarities and tendencies found.

### Descriptive analysis

#### Evolution of the number of academic papers

**Fig. 4:**
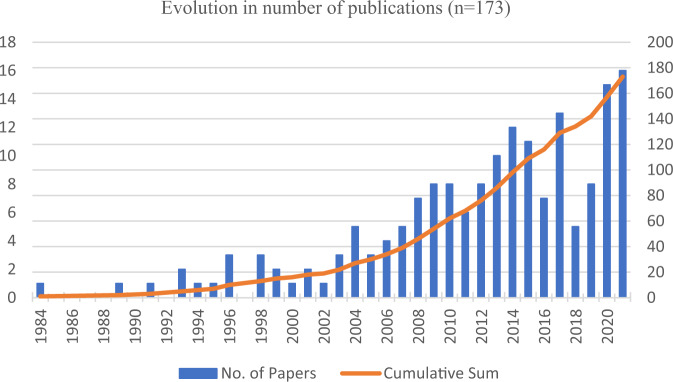
Year-wise evolution of the academic article on air transport and firm valuation. The figure shows the significant changes in term of the number of articles being published on the joint field of airline industry and firm valuation over time.

Figure 4 illustrates the evolution of the selected academic articles between 1984 (the year the first related article appeared in a database) and 2021. It shows that significant changes in publications took place, with ever-growing numbers of articles annually and an evolution in the publishing pattern, implying that interest in the topic has undergone significant changes. The upward trend is especially obvious between 2008 and 2018, with 91 of the 173 (52%) articles published in this period. The trend is even more evident in the last 3 years of the period, with 39 articles published. It is worth mentioning that scientific interest in the topic is in line with the ongoing status of global business. During the period 2008‒2018, the world was suffering and recovering from a financial crisis, making stock market volatility a popular topic. However, following the full recovery of the market in 2017, the issue of firm value became less significant, hence we can observe a decrease in the number of published papers in 2018 and 2019. The topic drew renewed attention in 2020 and 2021 because of the COVID-19 pandemic, which threw the airline sector into the darkest period in its history. With the sharp decline in demand and activity in the industry, airline values have fallen dramatically. In this context, the scholarly discussion to find managerial orientations that can ensure the protection of stakeholders’ wealth is unsurprising. In this regard, there is an urgent need for research to address the effect of support measures to overcome the crisis, such as suspending some business operations to reduce costs, relief on taxes and charges and the design of proactive strategies for governments to tackle the sharp changes in oil prices.

#### Most cited papers and sources

Some statistics from the selected papers are shown in Tables 3 and 4. Notably, we report the most cited publications and the most productive sources, obtained from the systematic review process. The number of citations illustrates the impact of all the articles, authors and journals. The idea behind the statistics is to assess which paper has received the most attention in academia by adding the number of citations for all the articles published by each author. We also report the most productive and the most cited journals.

**Table 3:** Top 10 cited articles.

Article		Year	No. of citations	Average citation per year
Author (s)	Title			
Kang et al.	Impacts of positive and negative corporate social responsibility activities on company performance in the hospitality industry	2010	312	26
Xie and Shugan	Electronic tickets, smart cards, and online prepayments: When and how to advance sell	2001	261	12
Hadavandi et al.	Integration of genetic fuzzy systems and artificial neural networks for stock price forecasting.	2010	248	20
Jerath et al.	Revenue management with strategic customers: Last-minute selling and opaque selling	2010	164	13
Carter et al.	Does hedging affect firm value? Evidence from the US airline industry.	2006	162	10
Drakos	Terrorism-induced structural shifts in financial risk: airline stocks in the aftermath of the September 11th terror attacks	2004	127	7
Behrens and Pels	Intermodal competition in the London–Paris passenger market: High-Speed Rail and air transport	2012	120	12
MacKerron et al.,	Willingness to pay for carbon offset certification and co-benefits among (high-) flying young adults in the UK	2009	114	8
Sun and Kim	Does customer satisfaction increase firm performance? An application of the American Customer Satisfaction Index (ACSI)	2013	83	9
Luo and Homburg	Satisfaction, complaint, and the stock value gap	2008	69	7

**Table 4:** The most productive and the most cited journals.

Journal name	No. of papers in the study	No. of citations	Impact factor	Quartile in Scopus
Journal of Air Transport Management	23	126	4.13	Q1
Transportation Research Part-E Logistics and Trans	7	31	6.87	Q1
The Service Industries Journal	5	3	6.53	Q1
Transportation Research Part-D Transport and ENVIR	4	13	5.49	Q1
Energy Economics	3	31	7.04	Q1
Journal of Financial Economics	1	115	6.98	Q1
The Journal of Finance	1	114	7.54	Q1
Journal of Marketing	1	41	9.43	Q1
Management Science	2	38	3.93	Q1
Review of Financial Studies	1	38	4.64	Q1

Tables 3 and 4 show that the most cited article is by Kang et al. (2010), entitled “Impacts of positive and negative corporate social responsibility activities on company performance in the hospitality industry”, receiving a total of 312 citations. The Journal of Air Transport Management is the journal with the highest number of publications, with 23 papers, and it also has the highest number of citations. This is to be expected since this journal is the specialised resource for all air transport issues. In terms of the number of publications, the next resource is Transportation Research Part E: Logistics and Transportation Review with seven publications, while the Journal of Financial Economics has the second-highest number of citations. These two journals issued 30% of the papers included in the sample, with the remaining journals publishing just one paper each, demonstrating that the sampled papers are not uniformly distributed among different journal publications.

#### Geographical scope

**Fig. 5:**
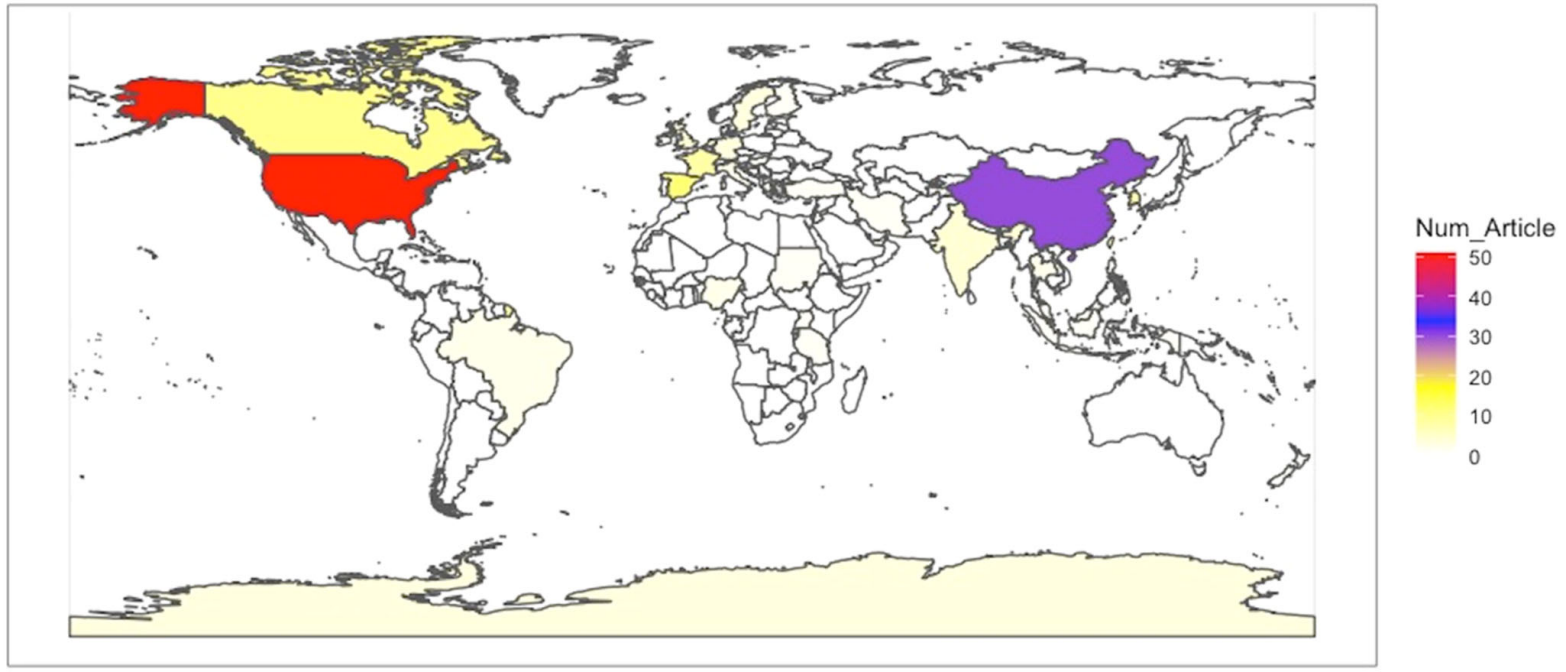
Geographical focus of authors by authors (RStudio). Spatial distribution of sampled articles showing that USA has been the most productive country.

We also analysed the authors’ country of affiliation to identify the spatial distribution of the present research topic. Figure 5 depicts the countries contributing to the topic, with the USA as the top contributor (62 papers), followed by China (30 papers). Spain (16 papers) is the third most interesting country on the topic of valuation in the airline industry. Based on the review, the topic is North America/Europe-centric, with 74% (129 papers) of attention to the topic coming from these two continents.

#### Co-authorship analysis among authors

Scientific collaboration could be defined as the cooperation that takes place within a social context between two or more researchers, which facilitates the sharing of meaning and the fulfilment of tasks relating to a mutually shared goal (Sonnenwald, 2007). In other words, co-authorship as an ongoing procedure could be characterised as separated segments that motivate people to exchange expertise, skills and information (Samitas and Kampouris, 2017). From an academic perspective, co-authorship advances innovation in knowledge transfer in the transition phase to an innovative partnership between universities (Chen et al., 2013), encouraging researchers to work together to intensify the quality and quantity of published articles (Samitas and Kampouris, 2017).

**Fig. 6:**
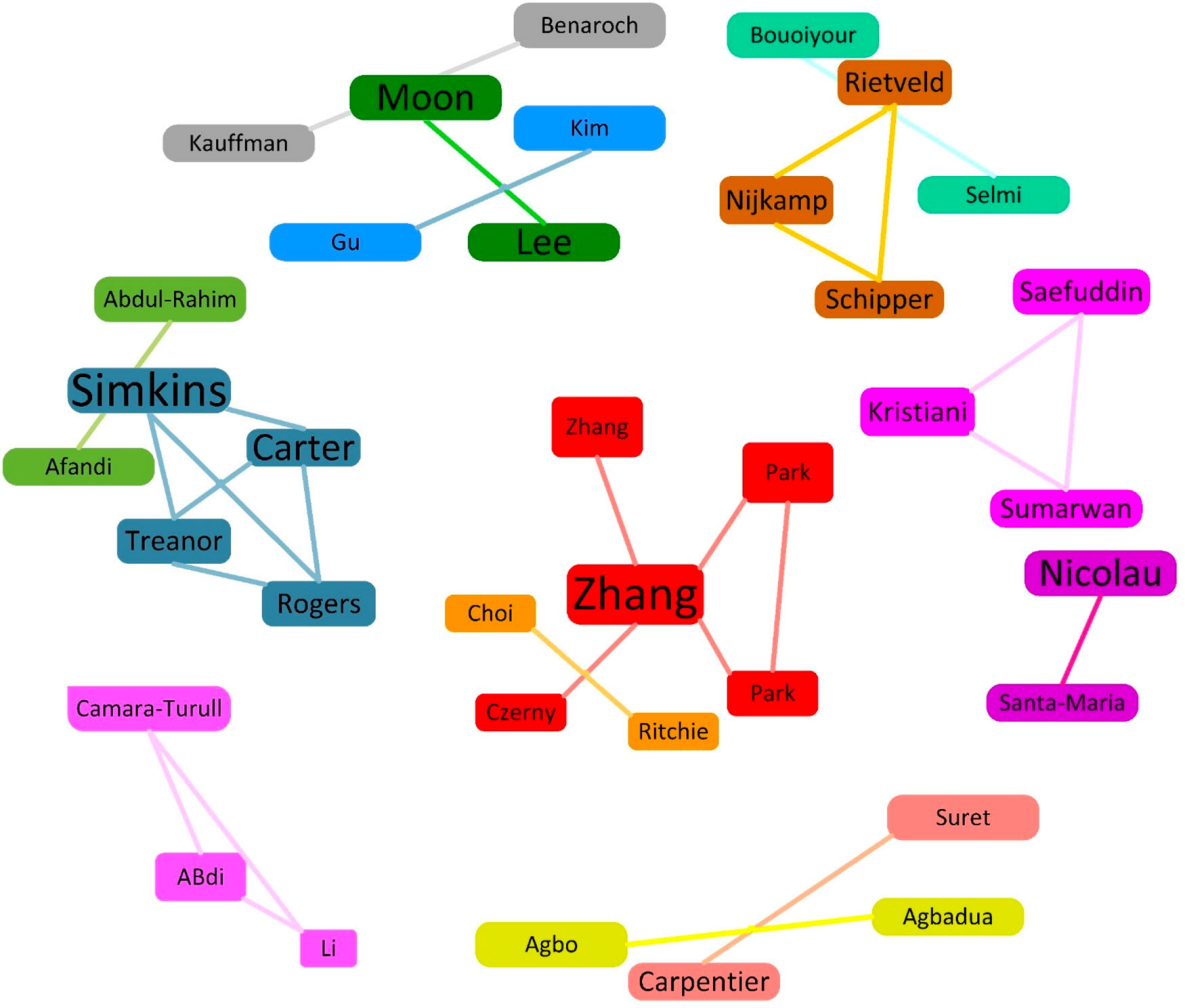
Co-authorship among the authors (adopted from the graph by biblioshiny package-R). The largest connected co-authorship network in the dataset, analyzed using VOS clustering. The figure shows a weak collaboration among researchers publishing at this field.

We used the full counting mode to identify the data selection and thresholds. To apply the method, we considered an author’s finite number of documents as 1 (minimum number of edges). Figure 6 shows the collaboration network of authors, illustrating the interplay between scholars in this field. In this figure, the number of publications by each team determines the size of the boxes. The distance between the two boxes is interpreted as an indication of the intensity of the relationship between authors (Shi and Li, 2019). The shorter the distance between two authors, the stronger they have co-authored with each other. When specific authors collaborate closely, their respective nodes are thicker and closer. Connected authors are commonly grouped together. For example, the cluster consisting of Zhang A, Hu Q, Zhang Y, Czerny A, Park J-H, Park N-K have collaborated closely, usually conducting joint research. Zhang A obtained the highest total link strength among the authors, having taken part in five research projects.

The size of the nodes in Fig. 6 is directly dependant on the number of publications, while the different colours connect the different authors. From the distribution of the clusters in the graph, we can conclude that the authors have a weak collaboration tie and are barely connected, possibly indicating that the researchers attach importance to establishing more collaborative relationships. To this effect, the information flow will have a higher propensity to diffuse throughout the field.

#### Keyword co-occurrence analysis

**Fig. 7:**
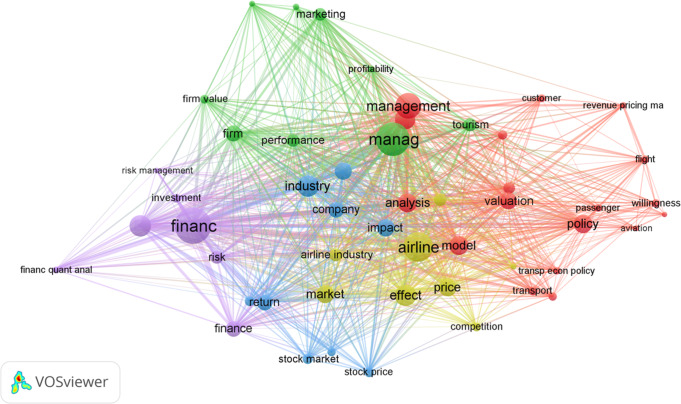
Keyword co-occurrence analysis (using VosViewer). The frequently co-occurring keywords, themes, or topics in research in the firm value at the airline context. The figure highlights three words “airline”, “finance”, and “managment” with the highest frequency in the sampled articles.

The ‘co-occurrence analysis’ provides the network of conceptual relations from the perspective of researchers in the field. By placing the words in context, and in relation to other terms and concepts, the co-word map can be seen as a semantic representation of knowledge structures (Tijssen and Van Raan, 1994). It involves the co-occurrence of words defined by the researchers in the articles, and those defined by professional indexers. The co-occurrence of keywords happens when two or more words appear together in a research study. Figure 7 (constructed by the VosViewer software) contains the keyword co-occurrence analysis of the terms *firm valuation* and *air transport*. Some points relating to the proximity between nodes, their size and the thickness of the lines between them must be considered to interpret the figure. Regarding size, the bigger the node [word], the larger the weight. The degree of relationship among the words is also shown by the distance between nodes. A shorter distance generally means a strong connection, while a thicker line reflects a greater co-occurrence between terms.

Sixteen main keywords with a minimum of four occurrences appeared on applying the co-occurrence network, highlighting the dominance of air transport and stock market terms. As expected, the word *airline* had the highest frequency among the analysed documents. The second most frequent concept among the sixteen terms was *value*, reflected in many forms such as firm value, stock price, stock market, price, valuation, market and performance. The collection of papers, therefore, stands out as a more cohesive body of literature when the subject is valuation. Accordingly, and from the joint analysis of the figure, readers can appreciate that *management*, *finance*, *airline*, *valuation*, *policy*, *analysis*, *industry*, *stock*, *analysis* and *return* are popular words when addressing valuation in the air transport context. These keywords are shown in five clusters represented by different colours. Specifically, *management*, *finance*, *airline industry* and *stock returns* are the most prominent keywords to represent the topic over the whole period. The involvement of keywords with higher centralities is because the issue dealt with by the literature is primarily a managerial one, and second because the subject is highly oriented towards the area of finance. Third, the academic interest focused specifically on the airline industry in the period was substantial, although this key term was less important than the two research keywords listed above (Management and Finance).

### Thematic analysis

Based on an initial reading of the 173 sampled papers, the influence of seven major groups of factors on airline value can be defined based on major themes. Notably, this categorisation is a purely manual classification of the sample since no coding method was used. It appears that the entire transportation industry is highly influenced by the impact of system risks due to a broad range of external factors, including financial events (recession, fuel price change, etc.), natural calamities (hurricane, tsunami, polar vortex, etc.) and man-made disasters (war, terrorist attack, etc.) (Deb, 2021). Almost all these studies use quantitative methods to approach the topic. This is not surprising since for our sample we tested two-factor associations (firm value and another quantifiable variable such as oil price, implementing sustainability standards, etc.) and it is a variance question in nature (Hermundsdottir and Aspelund, 2021; Van de Ven, 2007). Table 5 provides the detailed categorisation and references for the main research themes.

**Table 5:** Contents and references for the main research themes.

Topic area Industry-level characteristics
*Impact variable* Mergers and acquisitions (M&A), industry financial crisis, oil price, international connections, slot policy, crashes, how introducing low-cos carriers impact stock price of full-service airlines, alliance, aviatic innovation system (AIS), inter-industry competition based on announcements of new routes, what are the consequences of oligopolistic competition on spillovers in financial reporting, countervailing power mergers, market structure, strike crises, monopoly
*Method* case-by-case analysis, panel data, vertical-structured model, the so-called Fama–French–Carhart’s (1997), DHS model, event study methodology
*Main finding* *M&A*: It is shown that by adopting investment valuation and presenting innovative patents the more vulnerable company could achieve a favourable price when discussing for M&A deal. Also, lack of support for hypothesis that the merged airline could achieve countervailance against airports. *Oil price shocks*: Demand-driven oil shocks is proved to have an significant and positive impact earnings of airline companies. Also, it has been documenting that risk exposures of oil prices change significantly during the time. It is also found that airline business model (i.e. full-service and low-costs) is an important factor determining how it performs. Low-cost carriers are shown to have a more efficient stockpile method which provides some kind of insurance against on current jet fuel price volatilities. Therefore, the adjustment of firm’s product structure can improve its ability of resisting the crude oil price risk. *Announcing the slot policy*: When demand/capacity ratio is extremely low, the airline will consider slot hoarding behaviour. Taking hoard slots policy in form of operating excessive flights, needs to utilise specific options such as using smaller aircraft, charge a higher airfare, and provide services for more passengers. *Aircraft crashes*: Depends on the number of people within a particular crash, the respective airline will experience negative abnormal returns. However, the rival airlines’ stock prices will also suffer when the big tragedies occurred. Conversely, in the case of small-scale fatality crashes, rival airlines’ stock prices will appreciate. *Competition and market entry decision*: Market characteristics such as oligopoly structures, economic barrier to entry, and higher fixed costs are influential in making the industry more competitive and on network expansion in form of low-cost airlines’ entry. This could be explained by two possible factors: competition, and network expansion, for the effects of low-cost entry and that these have contrasting impacts. *Alliance*: Both alliance formation and termination are considered as two interrelated events to influence stock market. In such a case, it is complicated to reverse initial valuations before the alliance formation happen. Finding on alliance also suggest that degree of rivalry between the alliance partners and their rival airlines do not appear to moderate the effects on rival firms. This later result suggest that which international alliances could potentially improve partner firms’ competitiveness and their profitability, which in turn will cost firm value for rival companies. *Aviatic innovation system (AIS)*: AIS offer an airline with innovation-oriented techniques to create a future strategy. This way it is assumed the AIS can guarantee that the airlines will meet its obligations, likely to take a profit and therefore survive in near future. *Industry announcement of new routes*: Market value of firms in such a case are likely to both take some profits or fall. This depends on costs of launching for the communicator, and on the firm’s competitive power against the rivals in the market. *Unexpected earning announcement by rivals*: For an airline, reporting unexpected earnings by competitors lead to significant volatilities in its stock prices. Price volatility in this case depends on the extent of rivalry between the announcing and non-announcing companies. *Market structure***:** The impact of network will increase price competition. However, based on the size of the market and also on consumers’ valuation of waiting time, there would be various subgame perfect equilibrium configurations.
*Future studies* Future research should investigate how oil price shocks affect sector output, profits, or investment throughout the region. Also, since the factors presented could correlate, such interactions may be addressed by upcoming contributions. Additionally, to obtain a generalise image of the industry it is suggested to extend the analysis covering dedicated cargo airlines or combination carriers. This is because both groups of airlines may show similar or different characteristics. Finally, extending the sample and data on world-wide carriers is suggested. This is because studies presented are suffered from small sample sizes and therefore hesitate to generalise to the industry. Therefore, more empirical evidence is needed on evaluating the influence of industry-level value determinants for airlines.
*Study* Mohanty et al. (2014), Bernile et al. (2012), Detzen et al. (2012), Davidson and Worrell (1993), Bosch et al. (1998), Domke-Damonte (2000), Cheng and McDonald (1996), Cheng et al. (2021), Akyildirim et al. (2021), Mollick and Amin, (2021), Trifonov, (2021), Wang and Gao (2020), Thorbecke (2019), Sheng et al. (2019), Ho et al. (2013), Wassmer and Meschi (2011), Chen and Chen (2010), Alves and Barbot (2010), Cheng et al. (2009), Benaroch et al. (2007), Gong et al. (2008), Flouris and Swidler (2004), Kiesel et al. (2017), Gaudenzi and Bucciol (2016), Czerny and Zhang (2015), Kwoka and Gu (2015), Park et al. (2003), Ho et al. (2021), Yun and Yoon (2019), Venkataraman and Ramachandran (2016), Murphy et al. (2013), Hunsader and Dickens (2011), Murphy (2006), Park et al. (2003), de-Fusco and Fuess, (1991), Beneish and Moore (1994), Shepherd (1991), Allroggen et al. (2015).

Topic area Firm-level value influencers
*Impact variable* Business model (low-cost & full-service); airline’s distribution capability and retailing platforms to set offers to customers, firm demand for hedging, aeronautical charges, vertical differentiation strategy (which involves bear the cost to upgrade a base product to a premium version), cash compensation for chief executive officers (CEOs), new route announcement, service‐quality attributes in an airline choice, bankruptcy protection, flight and network efficiency, mobile app, financial and non-financial disclosure, leasing choices, oil refinery policy, strategic risk-taking behaviour of CEOs, bankruptcy, technical efficiency, discrete choice analysis, agency costs, sell the insurance, pricing policy, wage concessions
*Method* survey, panel data, real options valuation (ROV) approach
*Main finding* *Airline’s business model***:** It is shown that full-service airlines perform better than their low-costs counterparts when unexpected negative event happened and devastate the stock markets. *Hedging*: It is reported that hedging is positively correlated with firm value. Also, it is discussed that hedging could improve income predictability, operating performance increased for the affected firms, and increase (decrease) analysts’ forecast accuracy. It is also argued that airline companies may consider hedging their positions in jet fuel by applying a so-called horizon-sensitive model which is capable to directly accounts for jet fuel volatilities and crude oil spread. *Economy seating upgrades***:** Offering of economy travellers the chance to upgrade to higher class of service increases the price dispersion and revenues. *Route strategy*: Introduce a new domestic as well as launching multiple new routes lead to higher financial return for airlines. However, the way this advantage is obtained depends on sequence of announced routes in the way that early entrants gain more than later entrants. Moreover, offering a price-discounting strategy is related with market value of a firm. It is reported that specific expansion activities such as aircraft purchases or crew recruiting could remarkably provide the potential for higher revenues. *Mobile app*: A study reports that launching a mobile apps for lodging and airline companies could potentially promote their shareholder returns by 1.32%. *Leasing*: Operating profit of low-cost carriers are influenced more by leasing choices than that of their for full-services counterparts. In addition, when a low-cost airline deviates from the optimal level of leasing, it is subjected to bear more loses than a full-service one. *CEO***:** Airline’s strategic risk-taking strategy is considerably influenced by the tenure and education level of its CEO. *Bankruptcy***:** during its bankruptcy, the firm’s value will drop significantly. For example, Eastern airline’s value dropped over 50%. *Wage***:** Wage cuts positively impact the stock prices. However, the impact for cases of wage freezes or two-tier settlements are not significant.
*Future studies* Regarding the business model, it would be likely to explore how business model influence the rebounding of firm value after crisis and estimate the time to reach the value before crisis. Future contributions may also consider investigating the role of state intervention. Also, the factor being reflected in such a studies is that they are relatively suffer from small number airlines in analysis. This is an important factor because radical stock price volatilities of a single airline were able to move the average prices of the whole sample which is likely to create bias. New route announcement is a subject with high capacity to grow since very few studies have investigated it. Upcoming studies are advised to investigate the role played by factors such as competition mechanisms, shareholder base and governance on the market value of airlines. These factors also are likely to influence financial performance of airline companies. It is suggested to create a structural model for performance of air carriers by utilising data on competition at the route level, operators’ characteristics, airlines’ ownership, and governance indicators (number of independent directors, size of the board of director). This way, the analysis is likely to account for these effects and provide some more accurate predictions on individual optimal strategies to improve profitability. Finally, the inconclusive findings on the effectiveness of financial hedging implies the necessity of further empirical investigations to obtain a definitive conclusion for the aviation industry.
*Study* Kökény et al. (2021); Wang et al. (2021); Ranasinghe et al. (2021); Giambona and Wang (2020), Álvarez-Sanjaime et al. (2020), Cui et al. (2019), Hu et al. (2019), Bertus et al. (2009), Gu and Kim (2009), Tsai et al. (2008), Carlos Martín et al. (2008), Gong, (2007), Carter et al. (2006), Hung and Liu (2005), Chen et al. (2017), Qin et al. (2017), Bayer et al. (2017), Bourjade et al. (2017), Manuela et al. (2016), Lee and Moon (2016), Korkeamäki et al. (2016), Turner and Lim (2015), Berghöfer and Lucey (2014), Treanor et al. (2014), Swaminathan et al. (2014), Carter et al. (2006), Weiss and Wruck (1998), Alam and Sickles (1998), Singal (1996),Hersch and Mcdougall (1993), Ramanchi et al. (2017), Nwude et al. (2016), Kizildag and Goh (2011), Hofer and Eroglu (2010), Thomas et al. (1995),Özcan (2019).

Topic area Sustainability
*Impact variable* Environmental, social and governance (ESG), Sustainable Development Goals (SDGs), donation proposals (willingness to pay for sustainability initiatives), negative externalities caused by air travel, willingness to pay (WTP) a price premium for flights using bio-fuel blends, innovation (allocating funding for innovation activities bring potential fluctuations in the risk to an airline’s stock value), corporate social responsibility (CSR), voluntary carbon offsets, top management team (TMT), CEO apology (level of responsibility against unexpected negative event determine apology behaviour), innovations involving information technology (IT), environmental externalities
*Method* Panel data, Enhanced and Efficient Earned Value Management (denoted E^2^-EVM), real options methodology, contingent valuation (CV) method, double-bounded dichotomous choice of contingent valuation method (CVM), contingent valuation approach, stochastic dynamic programmes (DPs)
*Main finding* *ESG standards*: It is shown that implementing governance and environmental initiatives lead to higher market value for a firm. Additionally, the results for firm’s contribution to social pillar activities is disperse. The moderating role of firm characteristics (e.g. size, age, etc.) on the association between sustainability activities and firm value and performance have also been explored. *Willingness to pay for carbon offset*: Behavioural and characteristical features of respondents such as the quantity of CO_2_ reduced or offset by the initiative project, gender, education degree, occupational status, environmental consciousness, and travel habits will specify the passengers’ willingness to pay for carbon offset. It is suggested that uptake of voluntary offsets may be encouraged by investing in projects with co-benefits and by emphasising those co-benefits to consumers. *Apologising*: Level of responsibility against unexpected negative event also determines the stock price volatility level. *Innovations*: depends on the type of innovation, airline’s risk may rise via volatility. Among these innovative activities, advanced consumer segmentation innovations are shown to have a greater negative impact on sales than on fixed costs.
*Future studies* Testing for the potential moderation role of leverage, return on assets or dividends on the relationship between sustainable development initiatives and market value is likely to appear in future academic contributions. For willingness to pay, it is recommended to complement the data by the investigating the current voluntary contribution of passengers based on their observed behaviour. This is since the public awareness of global climate change has increased and probably the willingness to pay has gone to the same direction. This would lead to understand the reasons which are limiting the voluntary purchase of carbon offset units. One possible reason in this regard might be due to the inconvenience of paying the donation. The other potential explanation is due to the lack of properly designed proposals offered by airline firms. Also, considering inconclusive findings on the impact of CSR activities on the firm value, future studies may utilise a methodology that allows each CSR activity to have their own weight. It is also suggested to employ data that are more clearly and comprehensively encompass CSR domains. One possible method in this regard is to use structural equation modelling (SEM). The SEM is likely to offer a greater level of precision and depth in the analysis of the determinants and consequences of each CSR measure. In conclusion, more studies are demanded for: 1. Provide more evidence to reach the conclusive impact of each of these sustainability related items on firm’s performance and market value. 2. Quantify the exact impact of each sustainability undertakings on firm’s financial records. 3. Explore how firm characteristics could impact investment in sustainability and vice versa.
*Study* Abdi et al. (2021); Rambaud et al. (2021); Rotaris et al. (2020); Abdi et al. (2020), Racine et al. (2018), Shaari et al. (2020), Sonnenschein and Smedby (2018), Goding et al. (2018), Choi and Ritchie (2014), Nicolau and Santa-María (2012a), Nicolau and Santa-María (2012b), Azar and Johansson (2012), Lu and Shon (2012), Kang et al. (2010), MacKerron et al. (2009), Baarsma and Lambooy (2005), Lee and Moon (2018), Choi et al. (2018), Karaman et al. (2018), Kim et al. (2017), Lee et al. (2017), Moon et al. (2016), Choi (2014), Suksmith and Nitivattananon (2015), Kauffman et al. (2015), Carpentier and Suret (2015), Jou and Chen (2015), Schipper et al. (2001), Clarke et al. (1999), Corte and Gaudio (2014), Schipper et al. (1998).

Topic area Customer relationship and marketing
*Impact variable* Marketing productivity, demand type price sensitivity, security policy, willingness to pay (WTP) for business class seats, social media word-of-mouth (WOM), financial value of the frequent flyer members, third party customer complaints, passengers’ valuations on airline service attributes using stated preference analysis,
*Method* Survey, finite mixture structure individuals, panel data
*Main finding* *Dynamic marketing productivity (DMP)****:*** It is argued that DMP could potentially offer some competitive advantage for an airline. This benefit is gained via DMP’s positively effect on a firm’s financial performance and intangible value. *Demand type price sensitivity*: The findings reveals that there are two groups of travellers. The first group is high type “business” traveller which are less price sensitive and have a higher valuation and pays a higher price for services. The second group is low type “tourist” passengers with willingness to pay lower prices and consequently lower valuations. Also, it is reveals that when the departure date approaches, the proportion of high types also increases. *Security policy*: It is reported that people are willing to own full disclosure of information related with terrorist threats (including attacks on commercial airlines), regardless of the financial consequences for specific industries or future threats. *Customer satisfaction*: Studies concentrating on this issue show that customer satisfaction echoed in the profit margin, return on assets (ROA), return on equity (ROE), firm’s profitability, and in the market value of the service provider. For firms operating in the tourism and hospitality industry, there is evidence of positive affect of customer satisfaction on their revenues and market value. More specifically, if a firm gains more satisfaction rank, or if it introduces a new strategy to promote customer satisfaction, the firm may benefit from higher financial performance. *Service quality*: It is shown that airlines are incapable to maximise profits of service frequency, and that these firms are leaving the market. *Social media NWOM*: Studies on this topic show that there exists a mutual effect between social media negative word of mouth events promoted by users, and the effect that a piece of informational social influence has on the appearance of negative social media trends for a service. Findings also stress that service users compare a negative experiences and events for different brands in the market. *Financial value of the frequent flyer members*: If an airline develops an effective communication and cooperation with its customers, it is likely to maintain goodwill of customers and maximise its expected total returns. Findings related with this topic also suggest a considerable mutual benefits in introducing ‘voluntary overbooking’ policy that emphasises cooperation between travellers and an airline firm.
*Future studies* Company’s strategy to deal with its customers play a vital role in its longevity. In theory, satisfied customers can increase sales, reduce costs, consequently revenue for the firm and finally, improves firm value. Therefore, future research could reflect the reaction of customers in regard with the airline´s strategy or innovative activities. Potential studies also could take advantage of different customer behaviour track methods such as customer satisfaction score, net promotor score and social media modelling. Additionally, more contribution is demanded in regard with the social media since airline companies could connect with existing and potential customers, offer customer service, and demonstrate their brand. This is important due to the gap is evident in this area as currently there exist very few papers dealing with new social media potentials for airlines. Finally, consumers’ reaction to special events or crisis and their feedback on how management has been successful in controlling the situation related with the effect of the informational social influence, appears as an interesting topic for future work.
*Study* Escobari and Hernandez (2019), Rahman (2020), Smith et al. (2013), Sun and Kim (2013), Jou et al. (2013), Behrens and Pels (2012), Hess (2007), Huse and Evangelho (2007), Lijesen (2006), Nicolau and Santa-María (2017), Merkert and Beck (2017), Xun and Guo (2017), Janawade et al. (2015), Petrescu et al. (2020), Dalalah et al. (2020), Lu (2017), Kristiani et al. (2014), Jerath et al. (2010), Casado-Díaz et al. (2009), Jackson (2009), Chen and Wu (2009), Yang and Klassen (2008), Delquié (2008), Xie and Shugan (2001), Schwartz and Zea (1999), Lu (2017), Luo and Homburg (2008), Jiménez-Barreto et al. (2021).

Topic area International political and economic instability
*Impact variable* Political uncertainty, disasters arising from negative events such as terrorist attacks, Brexit, International regularity of open sky, deregulation,
*Method* Panel data, survey,
*Main finding* *Safety:* safety issues are directly associated with an airline’s earnings and market values. *Terrorist attacks***:** There is evidence of strong short-term effect on the valuation of airline firms when terrorist-attacks took place in Paris and Brussels. Furthermore, it is shown that smaller and less geographically diversified airline companies are significantly less affected by such a negative event. *Brexit***:** As the European Union had a central role in regulating various industries, vote to leave the Euro zone brought high uncertainties for UK enterprises. It is found that there is inconsistency between the adjustment of stock prices with the uncertain information hypothesis. Based on the evidence, policy changes are negatively correlated with stock prices. However, once the uncertainty-induced event is reduced, stock prices would increase again. Also there are other factors which are likely to negatively impact on UK industries. These factors include the lack of chance to benefit from the European passporting rules to set-up businesses, the opportunity to access to EU’s Research and Development funds, and the losing the chance to hire the skilled workers. *The global financial crisis*: It is claimed that the event significantly created the return’s volatility of airlines world-wide. The finding also suggest that major international events may all have risk effects on the airlines’ returns and stock prices.
*Future studies* Although, these critical situations cannot be controlled by airlines, future studies could focus on practical solutions to alleviate volatility in their returns. For instance, studies could measure the volatility in market value for airlines which has long-term agreement with jet fuel companies to control the extent of volatilities. Moreover, research could develop a review on airlines’ performance during political and economic crisis times to provide lessons from history and evaluate different strategies to better face these situations. Also, future studies are suggested to develop an alternative financial crisis and market value measures of airlines. Academic scholars may apply the dynamic conditional correlation model, which is likely to offer more policy insights. Another important issue is academic researchers may utilise different models to accounts for risk factors and potentially to figure out the optimal estimation laws.
*Study* Jeon (2021), Markoulis and Neofytou (2019), Flouris and Walker (2005), Kolaric and Schiereck (2016), Gillen and Lall (2003), Bouoiyour and Selmi (2018), Krieger and Chen (2015), Wang (2013), Rauh and Schneider (2013), Chen et al. (2010), El-Gazzar et al. (2009), Cam (2008), da Silva Rocha and Figueiredo Pinto (2006), Drakos (2004), Kim and Gu (2004), Goodrich (2002), Karels (1989).

Topic area New method to predict share price
*Methods* Component Analysis (PCA), two-stage (operational and stock market indicators) network data envelopment analysis process, the binomial option pricing model, Black-Scholes model, discounted cash flow (DCF) methodology, design a framework to account for acquisition and valuation risks, Multiple Objective Linear Programming (MOLP) optimisation model, integrated approach based on genetic fuzzy systems (GFS) and artificial neural networks (ANN), contingent claims valuation model, the real options framework to develop a multi-stage investment in the aerospace maintenance, repair, and overhaul (MRO) industry
*Main finding* It is claimed that introducing new model can help industry practitioners in figuring-out the profitability potentials. Also, new methods could be beneficial in complementing process of purchase or lease decisions of airlines. New models are of interesting for financial managers of airlines for several reasons. First, gain knowledge on how the capital markets value a firm, and verifying the existence and strategic importance of intangible assets are quite interesting for financial managers. It is because these managers are continuedly monitoring at the financial markets to figure out opportunities for future financing. Second, doing investigations on potential value creation of each project are necessary part for all major investment decisions. Findings of studies focusing on this topic provides insights on which factors will be considered by financial investors when they are making resource-allocation decisions. This factor is especially relevant to the airline industry financial managers because there have been remarkable historical changes during the evolution of the industry which changes its structure. Notably, following the global privatisation process, many airlines and airports are now operated privately, which normally rely on financial markets for provide funds for their projects. Furthermore, studies in this category explain theoretical reasons why and when firms can practice revenue management. Finally, new methods could help in investment appraisal decision making process in the industry. These methods are also beneficial in supporting capital decision making in the future.
*Future studies* Future studies are encouraged to apply feature selection with optimisation algorithms. Also, it is suggested to apply a mix of deep learning and SoftMax or support vector regression to address the issues related with market values estimations.
*Study* Zheng and He (2021); Zhang et al. (2021); Nikulina and Tarasova, (2021); Vasigh et al. (2020), Golbeck and Linetsky (2013), Malighetti et al. (2011), Ng (2007), Escobari (2014), Kalyebara and Ahmed (2012), Minja (2011), Hadavandi et al. (2010), Guzhva et al. (2010), Li et al. (2004), Miller and Park (2004).

Topic area Health crisis
*Impact variable* Covid 19, SARS
*Method* Event study, GARCH model, panel data,
*Main finding* Studies in this category are mainly to analyse the side-effects of the public health crisis on the stock markets. It is claimed that cost structure plays an important role in sensitivity of firms’ cash flows to lockdown. Although the negative event such as Covid-19, could severely devastate stock prices, it will not change the fundamental value drivers of firms. It is also claimed that firm characteristics such as size, leverage, cash flows, ROA, and more internationalisation degree play an important role in determining resistance level of firm to a negative shocks. For example, larger firms having greater cash reserves and higher market-to-book ratios experienced fewer negative returns, while firms having higher leverage levels were dropped more in value. In such a situation, the government would decide on whether economically support the firms (e.g., by providing cash or guarantee their obligations), or let the market mechanisms works no matter airlines file for bankruptcy. Given the importance of necessity to make decision by the policy makers, findings of these cluster of studies call for immediate reaction to minimise the negative impact of the Covid-19 in the airline industry.
*Future studies* Likewise political and economic crisis, critical heath situations are not controlled by airline companies. Future research could focus on developing a guidance for the investors on how to response to disasters. Further, more empirical evidence is demanded to design an intervention policy guideline for regulators, policy makers and industry practitioners in better react to events such as noble Covid-19 pandemic. The important aspect which has been reported as limitation of studies in this theme, is to strive to minimise limitations by developing new models and enlarge the sample to generalise the results. Future research is also encouraged to find out the manner stocks in different countries have changed because of the pandemic and try to explain cross-country differences in reactions based on each country’s industrial structures, its economic development category, and the scope of macroeconomic policy strategy to encounter the crisis.
*Study* Mohanty and Mishra (2021); Deb (2021), Singh and Shaik (2021), Thorbecke (2020), Carter et al. (2021), Maneenop and Kotcharin (2020), Liew (2020), Zheng and He (2021), Das and Mahapatra (2020).

#### Industry-level characteristics

We identified the subject of inconsistencies in industry-level performance, including mergers and acquisitions (M&A), aircraft crashes, alliances, inter-industry competition, etc., as the most popular topic to study the effect of these actions on firm value (37 papers). Some of these topics were widely covered in the sample. For instance, Wassmer and Meschi (2011) studied the impact of code-sharing alliance formations and terminations on the stock price of airlines, finding that the stock market reacts to these incidents. The next notable determinant is oil price volatility, which significantly impacts the operation of firms in this industry. Wang (2013) extracted three main reasons from the literature as to why the oil price is an important factor for airline firms. Their first two reasons are based on the discounted cash-flow model, highlighting a firm’s future cash-flow as a value influencer: (a) oil is an important natural resource in economic activities, influencing costs and expected cash-flows and (b) rises in oil prices leads to inflation. If the price increase is met by an anti-inflationary policy (i.e., a rise in interest rates) from the central bank, higher interest rates cause discount rate incensement, which ultimately has an adverse effect on the stock price for the firm. The third reason is that a rise in oil prices will increase commodity prices, which will ultimately lead to diseconomies of scale. In all cases, oil price increases magnify the operating costs for airlines and reduce their profits (Mollick and Amin, 2021). Therefore, there is a connection between oil prices and stock market returns for airlines. Furthermore, and specifically, within this category, factors such as competition, co-specification, merging, aircraft crashes, bankruptcy, accidents and the market structure have been mentioned as value influencers, matching the theory discussed by Malighetti et al. (2011). These contributions provide insights into the association between asset prices and changes in these value driver factors, which may be of interest to researchers, industry practitioners, financial managers and decision-makers. Large-scale transport for long-distance travellers has tended to be seen as a cost-benefit calculation for policy recommendations (Kristoffersson et al., 2021).

#### Firm-level value influencers

Studies in this category mainly analyse changes in firm-level performance, including an airline’s business model (low-cost and full-service), firm demand for hedging, new route announcements, bankruptcy protection, etc., as the second most popular topic to study the effect of these actions on value (36 papers). For example, Kökény et al. (2021) explore whether the stock market performance of European airlines influences their business model, finding that an airline’s business model provides insight for investors into what type of market reactions can be expected in the various stages of an operation. This also enables investors to utilise appropriate criteria and financial metrics, while making investment decisions. This means that full-service airlines show significantly better performance than their low-cost carrier counterparts when crises hit and stock markets are devastated. This hypothesis gains support from Deb (2021), who documented that two small-scale services Compass Airlines and Trans States Airlines, as well as Virgin Australia, filed for bankruptcy due to the COVID-19 pandemic. Also in this category, factors such as flight and network efficiency, launching a mobile app, leasing choices, oil refinery, CEOs’ strategic risk-taking behaviour and technical efficiency have been mentioned as value influencers, which are matched with the theory discussed by Malighetti et al. (2011). For example, in an interesting study, Manuela et al. (2016) investigate Delta Airlines’ oil refinery acquisition strategy to hedge against rising fuel prices and its effect on its financial and operational performance. The results of the study indicate that the strategy positively impacted Delta’s income and that this was rewarded on the stock market via higher share prices following the acquisition announcement.

#### Sustainability

Many studies (31 out of 173) evidence the association between sustainability activities and firm financial performance and value. The articles with a *corporate social responsibility* theme bring together topics related to concerns arising from issues linked to firms’ environmental, social and governance responsibility. Due to the heterogeneity between terms referring to environmental, social and governance issues, in the present paper we use the term *sustainability* to represent these issues. Over the last few decades, the topic has not only gained interest among academic researchers, industry practitioners and investors but also among policymakers. It has been described as a voluntary corporate commitment to operate aligned with broader society’s expectations, rather than just traditional profit-making corporate behaviour (Casado-Díaz et al., 2014). This means that firms are encouraged to contribute to the sustainable development goals (SDGs), which could be achieved by developing strategies that integrate sustainable practices into their normal daily operations, with the aim of reaching their own sustainability (Escrig-Olmedo et al., 2019). This corporate responsibility is described as activities to proactively contribute to the sustainability agenda from all its financial, environmental and social perspectives. The corporate sustainability domain also covers a firm’s internal business operations and productions, management and strategy, organisational units, and marketing and communications with its stakeholders (Escrig-Olmedo et al., 2019; Lozano, 2015). These studies link a firm’s financial performance and value and the level of commitment to sustainability standards in terms of professionally managing issues such as resource use, emissions, innovation, the employee-shareholder relationship, management and the board. These studies suggest that sustainability issues influence a firm’s market value as well as the financial performance of air transport companies (Abdi et al., 2021), which will be consequently reflected in the stock market.

#### Customer relationship and marketing

The next sub-group focuses on the relationship between airlines and their customers. Consumer behaviour has long been analysed in the economic literature, appearing first in the transportation literature at the beginning of this century (Pan and Zuo, 2020). For airlines, consumer behaviour and especially the behaviour relating to airline choice is considered an important element in planning and is a basis for their strategies (Munoz and Laniado, 2021). This research strand emphasises the significance of managing the association with customers and its influence on firm value. Given the highly competitive nature of the global airline business, airlines implement a range of actions to satisfy and stay connected with their main customers, the passengers. These activities include marketing strategies (e.g., from social media activities to internal operations to improve safety and service quality). Based on the findings of these studies, market value modifications are expected due to changes in the level of passenger satisfaction, investment behaviour tendencies and wage concessions (Sun and Kim, 2013b). Operators and practitioners must consider these dimensions and attributes because these items are influential to the overall perceived quality of a firm’s services (Ojo, 2017).

#### International political and economic instability

Changes in the political and economic situation significantly affect the market value of airlines. We use political and economic instability as an umbrella for studies in this domain since changes in firm value are rooted in the current political and macroeconomic situation. On this basis, this stream of literature mainly discusses contributions to a firm’s market value and the reaction to volatility due to worldwide political and economic factors such as terrorist attacks, and the transmission of these shocks to the market. For instance, although oil price volatility is an economic phenomenon, significant changes in supply and demand are usually triggered by political disturbances such as terrorist attacks in oil producers or importer countries (Mollick and Amin, 2021). Although shocks of this nature affect various industries at both country and international levels, they are disproportionately felt by insurance companies and tourism (including airlines). The issue was well reflected by IATA director and CEO Pierre Jean Jeanniot a year after the 9/11 terrorist attack when he said that “we have lost more in a year than we have made in our entire history. This is an industry that is now in a deep hole. We must start looking for footholds and ways to climb quickly out of the financial abyss” (Drakos, 2004). These papers mainly reflect the increased uncertainty of the industry following such incidents. For instance, Gillen and Lall (2003) studied how trade linkage and airline alliances are important in the transmission of global economic shocks to market value, finding the negative impact of shocks such as the 9/11 attacks on the mean abnormal returns of airlines.

#### New Method to predict share price

Apart from the studies having internal and external value drivers in focus, there are contributions that introduce new methodologies to increase the prediction accuracy of stock prices. Notably, most of the studies in this sub-category could fit in the internal factor category, but since they spotlight the innovative aspects of introduced stock price forecasting rather than the correlation between specific variables with firm value, we decided to present them in a new category. Since stock exchange mechanisms are complex and the market is influenced by seasonal factors, it has been proposed that multiple components are likely to impact the estimation of models, including financial data, the way to extract those data, optimisation algorithms and prediction model parameters (Zheng and He, 2021). Therefore, the selection of model features could consider both technical and fundamental characteristics. To this effect, when the share price is stable attention is directed to technical features. However, in times of high fluctuations, fundamental features may be a priority. Therefore, using long-term historical data is suggested for operating airlines since they are likely to produce more accurate analyses (Zheng and He, 2021). The models developed in this category could enhance the accuracy of a firm’s valuation and assist investors in making timely decisions for their financial strategies and business operations.

#### Health crisis

The next factor influencing the market value of airlines was found to be the global health crisis. Severe acute respiratory syndrome (SARS) and COVID-19 are contagious diseases that threaten human life. Risks like these disrupt business operations in infected countries, irrespective of the industry. Given the contagiousness of COVID-19, infected countries adopted various measures to limit contact (such as stopping unnecessary movement outside the home and public transport, closing schools and universities, and strict social distancing measures). Restrictions such as these immediately affected the economy, with the airline industry the first to suffer due to the dramatic drop in passenger demand. Because of the COVID-19 outbreak, the market value of airlines shrank significantly (Maneenop and Kotcharin, 2020). Studies in this cluster mainly use the event-study model to analyse the influence of disasters of this kind on online stock prices. This method is popular in economics and finance for investigating the effect of news related to a particular event on stock market prices (Maneenop and Kotcharin, 2020).

By contrasting the situation before and after the outbreak, and the stock returns for the airline industry and the whole market return (Maneenop and Kotcharin, 2020), these studies investigate the extent to which the firms in this industry may suffer (Liew, 2020), how severe the impact is and what the impact on stock price volatility may be (Deb, 2021). Different events and daily data sets were selected in these studies, including crucial announcements such as days-from-first-case reports by China (January 13, 2020) and the USA travel ban announcement by President Trump (March 11, 2020). The findings of these studies are also interesting. In this regard, Liew (2020) observed the rapid decline in profit of airlines and tourism-related businesses by monitoring statistics derived from three leading consolidators, namely hotel accommodation, airline tickets and travel service services. Deb (2021) finds that COVID-19 had an unprecedentedly severe effect on the stock price movements of airlines. This author further proposed a method to predict the market reaction to similar events, especially in the short term. Last, Maneenop and Kotcharin (2020) find that airline share prices reduce more significantly than the whole return of the market. All three studies resulted in major changes in airline valuation theory. To this end, it is necessary to design a strategy to alleviate the economic side effects of the pandemic in the airline industry.

## Summary, implications, and future research avenues

Through the categorisation of value determinants of airline companies, the current study provides an approach to linking the theoretical concepts and practical findings by structuring them into seven main subject areas. In summary, we find that the topic is especially in demand as a result of the COVID-19 pandemic, which has driven a radical shift in scholarly productions focused on the value issue for airlines. Looking at the sources of publication of these papers, the Journal of Air Transport Management (with 23 papers and 126 citations) is shown to be the most frequent and the most cited journal. The geographical scope analysis for authors’ affiliation showed the USA (62 papers) as the highest contributing country, followed by China (30 papers). As far as co-authorship among authors is concerned, Zhang A et al. were the biggest cluster of authors working closely together. However, most authors in this field tend to work separately. The analysis of keyword co-occurrence indicates that, as expected, *airline* is the most cited keyword among terms in this context.

Regarding the thematic analysis, we find that the largest group of studies examines the industry and firm-level factors. The third largest group of papers focuses on modern sustainability initiatives and relations with firms’ financial performance. Based on the findings of these studies, a drastic stock market reaction is to be expected as a result of any changes at the level of airlines’ corporate, environmental, social and governance responsibilities. The next sub-group is composed of studies that examine the impact of changes in the political and financial status of an airline on its market value performance. These contributions suggest that variations in factors such as political instability, terror attacks, oil price shocks and jet fuel prices may lead to market volatility. Issues related to the customer and marketing strategy, health threats and firm-industry level value determinants were also found as main themes in our dataset.

By analysing themes, we found that there is evidence of a shift in academic contributions to sustainability initiatives and their consequences for stakeholders’ value because the airline industry is regarded as one of the most challenging when it comes to environmental impact and sustainability issues (McManners 2016a, 2016b). However, this finding does not correspond to reality. According to Heeres et al. (2018), only 38% of the top 100 airlines publish their sustainability report. This may be due to uncertainty as to whether environmental sustainability is compatible with financial sustainability.

Regarding passenger issues, an interesting finding is that customer satisfaction has grown since the idea of low-cost airlines has become widespread, especially in the new millennium. As would be expected, following the deregulation of the industry, more commercial and market-leading perspectives became the norm, and knowledge of passengers and their preferences gained interest in academia (Spasojevic et al., 2018). There has also been a noticeable shift in scientific articles centring on the value issue, especially since 2020 with the output of COVID-19-related academic publications, totalling 31 (over 17% of sampled papers). Such considerable academic attention stands out in the tourism and hospitality field, including airlines (Chen et al., 2022). Studies in this domain have highlighted the need for policy designs to alleviate the impact of the pandemic on the airline industry as the one most damaged by COVID-19. However, the literature has failed to conclude with tangible practical solutions to protect the value when a negative event occurs.

### Study implications

This paper contributes to the knowledge surrounding firm value determinants from both academic and industry perspectives. In terms of the first, despite the importance of valuation issues for firms, contributions to the tourism literature (including the airline context) remain scant. Considering the lack of review works as a response to an apparent gap, our study approaches the issue objectively by collecting the data from reputable journals in the WoS Core Collection-Clarivate & Scopus database. By doing so, we contribute to identifying and classifying the important value driver and influencer factors, helping to fill this gap and bring new insights by overviewing the relevant literature trends through the synthesis of the available documents. Additionally, a focus on the airline industry, which is one of the most important and rapidly growing industries, could contribute to the current body of knowledge with significant first-hand insights. To conclude, adding an in-depth systemic tendency to the wide divergent literature available in the field could benefit future researchers interested in air transportation business valuation and analysis.

The above consideration leads us to the second theoretical contribution of this study, which is the aggregation of existing knowledge on the topic of firm value in this context. Future researchers can find support for each of the concepts categorised among the analysed documents. The empirical findings confirm the theoretically anticipated firm-level financial and non-financial value drivers, together with external factors influencing the market value. This approach encourages researchers to recap understanding of the firm value topic through a new categorisation that can help to identify conceptual and empirical relations between main value-related subject areas. We observed that current contributions have turned their attention away from classic external and internal value drivers toward modern corporate social responsibility issues.

Systematic literature reviews can provide a reliable basis to formulate decisions and take action (Tranfield et al., 2003). In this regard, executives may also use the results to see how firm value issues are dealt with in the literature and benefit from the empirical results when designing business strategies and making decisions. Air carriers’ large-scale operations need a notable level of use of resources, and every major decision has an implication for a company’s financial returns, which will be reflected in its share value. This is understandable given the limited level of available resources for a company and the need to respect efficiency when allocating these restricted resources. Further, these companies should also bear in mind their competitors’ profiles and offering to customers (Chih et al., 2021). In practice, however, there is sufficient evidence of the inefficiency of airlines’ business strategy. To put this in perspective, Nzuva (2020) discusses how the majority of airline firms are suffering from low levels of profit, which hampers the industry from expanding. To this effect, business model modification is vital to meet airlines’ long-term growth.

Taking the above into account, we suggest that major changes in airlines’ value are caused by certain leading global trends such as the green finance discussion and unexpected negative events. Notably, the theme has gained momentum since the proposal of the 17 United Nations Sustainable Development Goals (SDGs) in 2015, which require allocating a firm’s resources to acquiring eco-friendly equipment, reaching higher standards for products and prioritising safety measures collectively as a corporation framework to “shift the world onto a sustainable and resilient path”. This trend has been even more in the spotlight since the outbreak of the COVID-19 pandemic since it induced a crash in firm value, causing stakeholders and institutional investors to look for sustainable profit-making shares and protect their wealth at this time of crisis. Consequently, airline managers and industry decision-makers have acknowledged the recent preferred shift towards the importance of sustainable development strategies to communicate their obligation to maximise the wealth of their stakeholders. This campaign towards sustainability also provides an opportunity to launch a sustainable development agenda, which acts as an insurance link against unexpected negative events such as health crises (e.g., the current pandemic and SARS) and international political and economic instability (e.g., the 2008 global financial crisis, terror attacks such as 9/11, among others). In this regard, our findings provide insights for managers who are considering allocating available resources to sustainability activities by adopting more efficient and robust approaches, which by comparing stock returns for the airline industry with the whole market return (Maneenop and Kotcharin, 2020) consider the firm’s characteristics in terms of its business model and ownership structure. For instance, in terms of investment in renewable resources, recent developments in technology may throw up potential opportunities to reduce energy consumption by utilising more fuel-efficient aircraft technology and introducing direct flight pattern networks. We suggest that managers consider these factors to act proactively under economic turbulence rather than take a reactive approach to crash value at times of crisis. This could also apply to potential policymakers, requiring firms to invest more in such initiatives to be beneficial not only for the firm but also for society in the long term.

### Limitations and future research

The selective, observational, and retrospective essence of this systematic review had several limitations. First, the search terms used cannot be assumed to be fully comprehensive and capture all the relevant academic articles. This is because a broad range of keywords has been used by researchers in the literature. We restricted the search to the definite and most probable keywords to capture the most relevant studies, making it almost impossible to cover the state of the field over time in a single study. To address this issue, future research could use a literature-exploration algorithm to find an almost overwhelming number of matching documents on a research topic. The second limitation is that we considered only articles published in the WoS Core Collection-Clarivate and Scopus databases. Future reviews should include articles published in other databases such as journal citation reports (JCR).

Third, within the papers found in the review, several studies were recognised as directly unrelated and removed from the study. Future studies may need to broaden the scope of the investigation in this regard. These studies could be improved by the investigation and assessment of advanced metamodels from other contexts and compliance with new techniques to be used (Binsuwadan et al., 2021). Additionally, due to the outbreak of COVID-19 and the questions it has raised about valuation, contributions to measure the effectiveness of preserving actions to survive the crisis, such as cutting capacity to reduce costs, relief on taxes and charges by governments, and to propose proactive strategies for policymakers to deal with fluctuating oil prices, seem necessary. In particular, a greater focus is needed on investigating the effectiveness of fiscal policies to prevent exposures in oil-related sectors such as the air transport industry. Furthermore, given that most studies in the literature concentrate on one or just a few airlines, more studies could be carried out using larger samples that cover a variety of firms. Last, the findings of the theme analysis may encourage more research on sustainable value drivers as a promising area of research. Additional contributions to provide significant information to understand the sustainable development agenda in recognition of firm value are needed for a sustainable future of the air transport industry.

## Data Availability

The data that supports the finding of this study are openly available in Scopus and Web of Science core-collection Clarivate databases. We used the articles published in these two databases through the right of access by the authors’ institution. These data are also publicly available from Google searches.
